# Analysis of burnout and its influencing factors among prison police

**DOI:** 10.3389/fpubh.2022.891745

**Published:** 2022-09-13

**Authors:** Jin Gao, Xinyang Du, Qing Gao

**Affiliations:** School of Humanities and Laws, Northeastern University, Shenyang, China

**Keywords:** prison police, burnout, influencing factors, job satisfaction, emotional exhaustion, negative detachment, self-efficacy

## Abstract

**Background:**

Burnout among prison police is an occupational health issue in the field of public health. Although burnout has been a hot issue for decades, there has not been a focus on the specific group of prison police. This study explores the burnout status and its influencing factors among prison police.

**Methods:**

The Maslach Burnout Questionnaire—General Survey (MBI-GS) was used to conduct a questionnaire survey among 1,024 prison police.

**Results:**

It indicates that emotional exhaustion, negative detachment, and self-efficacy were the most significant dimensions of the burnout among prison police officers. The results of multiple linear regression analysis showed that gender (−0.201, *P* = 8.8958E-11 <0.05), workload (−0.441, *P* = 1.6287E-9 <0.05), whether they have direct contact with supervisory subjects (−0.394, *P* = 2.1449E-39 <0.05), and a sense of organizational support (−0.298, *P* = 3.7182E-7 <0.05) were risk factors for burnout in prison police.

**Conclusions:**

Burnout among prison officers can be reduced through preferential treatment of prison police, sound organizational mechanisms, and self-improvement of prison police.

## Introduction

### Background of burnout in prison police

Prison police are responsible for execution of penalties, managing prisons according to the law, punishing and reforming criminals, preventing and reducing crimes, and maintaining social stability, which is the fundamental guarantee for the development of prison undertakings (1). From the perspective of group mental health, compared with other professions, prison police are subject to more work pressure, misunderstanding, risk, and unforeseen emergencies (2, 3). Long-term, high-pressure work tends to cause different degrees of burnout among prison police, which is not only detrimental to their physical and mental health, but also to their work efficiency and quality as well as the results of offender rehabilitation (4). At present, there are relatively few studies on burnout in prison police; most of the studies on burnout focus on bank employees, health care workers, and teachers (5, 6). Therefore, we focused on studying burnout in prison police, which would expand and enrich the research in the field of burnout, specifically in prison police. Based on available literature, we investigated and analyzed the current burnout situation among prison police by using a widely accepted theory and a fine-tuned burnout measurement questionnaire suitable for prison police, and conducted statistical analyses of the obtained data. The research question of this paper is: which factors affect job burnout of prison police, what is the influence degree of each factor, and what actions should be taken to avoid job burnout of prison police? This study takes prison police burnout as the research target vehicle to achieve the purpose of practical exploration of special occupational public hygiene health.

Burnout of prison officers may be influenced not only by individual characteristics, occupational stress, and perceptions about the organization, but also by overarching factors that reflect the characteristics of the prison environment. Studying burnout among prison officers is beneficial for them to work in healthier and lower security risk conditions (7). The study is not only related to the physical and mental health of prison police, but also to their stability as a team and the maintenance of social order in prison system. By studying the current situation and the influencing factors of burnout of prison police, we can further improve their efficiency, propose countermeasures to cope with the burnout, and help eliminate the negative effects of burnout on them. This study provides constructive references for improving the psychological health of prison police, reducing burnout, and improving work efficiency.

The significance of this study is that it focuses on the factors influencing burnout in a specialized group of people, the prison police officers. Due to the nature of prison police work, the subjects they work with are complex and socially harmful, the work environment is relatively closed, the work content is boring, and the working hours are long and stressful. Therefore, prison police officers are more prone to burnout than ordinary police officers or people in other occupations. The study also analyzes demographic influences and is more informative in terms of self-imposed content (8). Furthermore, it fine-tunes the Maslach Burnout Inventory—General Survey (MBI-GS) scale on the basis of existing literature on the influencing factors of burnout. It prepares a burnout questionnaire more suitable for the actual work situation of prison police officers by combining existing literature and the results of analysis of survey data using the theories related to burnout and empirical surveys. The current situation of burnout and its influencing factors for prison police was investigated from all aspects. The study revealed the pattern of burnout among prison police officers, and proposed countermeasures and suggestions, which provided a reference for prison management. The data obtained in this study are real and valid, which expands the research field of burnout and provides reference for future research on burnout of prison police.

### Review of occupational burnout literature

Burnout was first proposed by the American clinical psychologist Freudenberger (9). Maslach defined burnout in detail from a psychological perspective in terms of three dimensions: emotional exhaustion, depersonalization, and low personal fulfillment (10). According to Pines and Aronson, burnout is a subjective experience that includes a state of physical, emotional and psychological exhaustion, caused by being in a state of prolonged emotional need, which generally comes from one's own expectations and stresses (11). These conditions can lead to negative effects on one's work and life. Leiter argues that burnout comes from the gap between personal expectations to achieve a professional role and the actual situation of the organizational structure, and that it is a manifestation of emotional exhaustion (12). Hobfoll argues that there are three main manifestations of burnout among workers: emotional exhaustion, depersonalization, and a diminished sense of personal accomplishment (13). Moreover, studies have concluded that job stress has consistently increased among police officers in the last decade (14). Thus, burnout may be a long-term process of resource depletion and inadequate response to chronic work stress (15). Aljabr argues that identifying the demographic and occupational influencing factors of burnout during the COVID-19 pandemic helps plan psychological support strategies (16).

There are other issues that are relatively less studied, which directly lead to the increasingly low motivation and gradual loss of enthusiasm and vitality in work. Carbonneau and Vallerand found that in the face of heavy workload and work pressure, employees might feel physically and mentally exhausted due to the conflict between work and other activities in life; therefore, gradually lose interest in work, which could lead to burnout (17). Karasek proposed the Job Demand–Control model to study job stress (18). Another Job Demand–Control–Support model was also proposed, which provided a model for the broader perspective for study of burnout (19).

Yang pointed out that the level of burnout among prison police officers was highly related to their gender, age, income, marital status, time of joining the police, education level, and position (20). Morgan found that the effects of age and other demographics (e.g., gender, work experience, etc.) might contribute to prison officers' burnout (21). Kop found through a survey that prison police officers had a higher level of burnout compared to other professions in the Netherlands, and the main factors leading to this were lack of rapport with offenders and less communication with family and friends, which to some extent could affect the regularity and rationality of prison police enforcement (22). Martin and other scholars found that burnout among police was strongly related to citizens' lack of understanding and rough resistance to police enforcement (23). Among a range of police stressors, work-family conflict was a significant predictor of turnover, while burnout mediated the relationship between stressors and turnover intentions, while job satisfaction did not (24). Moderated mediation analysis showed that high job demands were indirectly related to high CWB, with job burnout acting as the mediator (25). Beata examined the relationship between prison police officers' emotions at work and burnout through an analytical study, and found that prison officers with a strong and positive work ethic were less likely to experience burnout (26).

By reviewing the literature and comparing the causes, influencing factors, and measurement tools of burnout, a set of mature burnout scales had been formed through the design and modification of burnout scales (27, 28). Research on burnout among prison police can be traced back to the 1980 s, and scholars have put forward many research-worthy views on the causes and countermeasures of burnout (29–31).

The existing literature has not paid sufficient attention to the factors influencing burnout among prison officers and has not established a framework for comprehensive analysis (32, 33). In this study, a lot of importance has been given in reviewing the existing literature (a). The theories established in existing research have been adopted and improved upon by this study. Its novelty lies in that it explores the critical topic of burnout at the workplace of a specific group of people—prison police officers. (b) The study contributes to existing literature by enriching it based on the analyses of the survey responses.

The hypotheses of this study are: (a) workload, work environment, and contact with criminals have an effect on burnout; (b) unfair treatment and lack of promotion may deepen burnout; (c) little personal time and lack of socialization affect burnout. Through empirical study, the main influencing factors and dimensions of burnout caused by different individual characteristics of prison police officers are identified and their causes are analyzed (34). This study provides a theoretical basis for the improvement of burnout among prison police through data analysis (26).

## Materials and methods

### Sample selection

This study was conducted after obtaining consent from superiors. Also participation in the survey was voluntary and anonymous for prison officers, and formal consent was obtained in all individual participants included in the study. This study took the prison police as the research subject, and focused on the current situation and influencing factors of burnout among prison police in Liaoning Province, China. A survey on burnout status and its influencing factors was conducted by randomly distributing electronic questionnaires within the prison police system in Liaoning Province, China, through the WeChat platform. In this study, data were collected by distributing an online questionnaire, and a total of 1,081 prison officers answered the questionnaire, and after excluding the unqualified questionnaires, a total of 1,024 prison officers were obtained, with a effective response rate of 94.8%. The samples with incomplete answers or those that did not match the content of the questions were excluded. The relationship between gender, marital status, age, years of work, mode of joining the police, Positions, education level, monthly income, contact with criminals, and overtime duty among the demographic variables and burnout was examined. Among those surveyed, 67.77% were male and 32.23% were female prison officers. The higher number of male prison officers was due to the special nature of prison work. This is consistent with the current situation of prison police in Liaoning Province, China. Overall, the demographics of the sample in this survey are consistent with the actual situation of prison police and the results of the survey are reliable. See **Appendix 1** (**Table A**) for the sample demographics.

### Methods

#### Population, study design, and study procedures

The study focused on the current situation and influencing factors of burnout among prison police in Liaoning Province, China. Therefore, a questionnaire assessing the factors influencing burnout in previous police was prepared. To do so, previous literature on the factors influencing burnout by domestic and scholars was combined, and the MBI-GS scale was fine-tuned according to the current actual situation of prison police. A total of 1,024 valid responses were received after preliminary screening and elimination of the unqualified ones. The required data were compiled and statistically analyzed using SPSS software, providing an important basis for the subsequent analysis.

The MBI-GS was used as the theoretical basis to conduct pre-interviews with 1,024 prison officers (35). The original questionnaire was combined with the actual situation of the prison officers to form the Burnout Questionnaire for Prison Police. Informed consent was obtained from the participants. The questionnaire comprised the three sections indicated below.

#### Demographic information

Demographic information included gender, marital status, age, years of service, mode of entry, Positions, education level, personal monthly income, situation of whether direct contact the of supervision during supervision, and current overtime or night shifts at work.

#### Burnout scale

This section included 20 questions involving the three dimensions of emotional exhaustion (questions 1–5), negative detachment (questions 6–12), and self-efficacy (questions 13–20). The questionnaire was rated on a 5-point Likert scale, with 0 indicating never and 4 indicating every day or more, and the higher the score the higher the burnout.

#### Burnout influencing factors

The Burnout Influencing Factors questionnaire on the factors influencing burnout in prison police consisted of 8 questions, of which questions 1–3 were on work level, 4–6 were on organizational level, and 7 and 8 were on personal level (36, 37). See **Appendix 1** (**Table B**) for specific questionnaire content.

### Statistical analysis

Statistical analysis allows for adjustment for repeated measures (38). It consisted of a descriptive and inferential analysis resulting in better burnout measurement results (39). IBM SPSS version 23.0 was used for statistical analyses. First, one-way descriptive statistical analysis, *t*-test, and variance test were conducted. Then, multiple linear regression analysis was performed for the study of influencing factors, with response variables of emotional exhaustion, negative detachment, and self-efficacy scores and total burnout scores. Dummy variables were set for unordered multi-categorical independent variables (such as interpersonal relationships, workload, work environment, and promotion).The dependent variable analyzed is a single composite measure of burnout. The reason is that the separate burnout dimensions can hardly comprehensively reflect individual prison officers' degree of burnout. Moreover, there was an inaccuracy in measuring with separate burnout dimensions, with some individuals scoring 0 on the separate burnout dimension score items. The composite measure of burnout contains the three dimensions of emotional exhaustion, negative detachment, and self-efficacy, and also integrates demographic information. In addition, we conducted model calculations with a composite measure of burnout (total burnout score) as the dependent variable.

## Results

### Quality analysis of the survey questionnaire

#### Reliability analysis

There were 20 items in this questionnaire to examine the burnout situation among prison officers, among which questions 1–12 and 13–20 were positive and negative option questions, respectively. There were 8 questions to investigate the influencing factors of burnout on prison officers, of which the first and third were reverse-option questions. All 28 questions were answered on a five-point Likert scale. In order to better measure the reliability of this questionnaire, the reverse-option questions were modified into positive-option questions.

In this study, reliability of the questions in each dimension of the questionnaire was analyzed for 1,024 responses. The results of the analysis are shown in Table 1. From Table 1, we can see that the reliability of each dimension is above 0.7 except for the personal dimension, which has only 2 questions and a reliability coefficient of 0.580 which is close to 0.6. This indicates that the questions of all dimensions, including the personal dimension, have good reliability and reflect the true extent of this questionnaire. The overall reliability of this questionnaire is 0.953, which indicates that the overall questionnaire has high reliability and stability, and truly reflects the consistency of measurement.

**Table 1:** Cronbach's Alpha coefficients for subscales and total scales.

**Dimension name**	**Cronbach α confidence coefficient**
Emotional exhaustion	0.906
Negative detachment	0.914
Self-efficacy	0.873
Work level	0.833
Organization level	0.739
Personal level	0.580
The whole questionnaire	0.953

#### Validity analysis

Validity analysis was conducted to test the construct validity, which tested whether the actual questionnaire structure was consistent with the expected structure. In this study, whether the index settings of the six-part variables were standard was tested. Construct validity is usually considered an effective way to evaluate validity. The results of factor analysis show that the test statistic of Kaiser-Meyer-Olkin (KMO) is 0.952 (Table 2). According to the metric of factor analysis, a value of 0.9 or more indicates a good fit (40). Therefore, the data in this case was suitable for factor analysis. Bartlett's sphericity test chi-square value was 22,839,776, corresponding to a *p*-value of 0.000, indicating that the current data were used after doing factor analysis. A total of six factors were extracted and the cumulative variance contribution rate was 74.677%, which far exceeded the criterion of 50% of the cumulative variance contribution rate of the extracted factors (Table 3). This coincided with the index system constructed by the questionnaire.

**Table 2:** KMO and Bartlett's test of the scale.

**The kaiser-meyer-olkin metric**		**0.952**
**of sampling adequacy**		
**Bartlett's**	**Approximate**	**22,839.776**
**sphericity test**	**cardinality**	
	df	378
	Sig.	0.000

**Table 3:** Eigenvalues, contribution rates, and cumulative contribution rates of each factor.

**Factors**	**Initial eigenvalue**			**Extraction of squares and loading**			**Rotate square and load**		
	**Total**	**% of variance**	**Cumulative %**	**Total**	**% of variance**	**Cumulative %**	**Total**	**% of variance**	**Cumulative %**
1 Emotional exhaustion	12.705	45.376	45.376	12.705	45.376	45.376	7.085	25.302	25.302
2 Negative detachment	2.602	9.292	54.668	2.602	9.292	54.668	3.586	12.807	38.110
3 Self-efficacy	2.047	7.310	61.977	2.047	7.310	61.977	3.207	11.453	49.563
4 Work dimension	1.560	5.570	67.547	1.560	5.570	67.547	2.793	9.975	59.537
5 Organizational dimension	1.181	4.218	71.765	1.181	4.218	71.765	2.361	8.434	67.971
6 Personal dimension	0.815	2.911	74.677	0.815	2.911	74.677	1.878	6.706	74.677

### Situation of burnout and its influencing factors

#### Descriptive analysis of the current situation of burnout among prison police

The percentage of people with high burnout in each dimension can be seen in Table 4. It can be seen that many prison officers showed high levels of burnout in the three dimensions of emotional exhaustion, negative detachment, and self-efficacy, with the highest number of people in negative detachment, followed by emotional exhaustion and self-efficacy.Based on the data shown in Table 4 combined with statistical analysis results, we obtained the overall situation of burnout among prison police.

**Table 4:** Number of highly job burnout in various dimensions.

**Dimensionality**	**High burnout**		**Moderate burnout**		**Low burnout**	
	**Number of people**	**Percentage**	**Number of people**	**Percentage**	**Number of people**	**Percentage**
Emotional exhaustion	312	30.5%	390	38.1%	321	31.4%
Negative detachment	399	39%	419	41%	307	30%
Self-efficacy	303	29%	460	40.5%	367	30.5%

The scores were calculated according to a 5-point Likert scale, and higher the score, the higher the degree of burnout, with 1–5, 6–11, and 12–20 being the emotional detachment, negative detachment, and self-efficacy dimensions, respectively. The value of one-third of the total score was used as the threshold value to divide them into three levels: high, moderate, and low degree burnout. It indicated that the Negative detachment dimension best highlighted a high level of burnout among prison officers, while more prison officers are at moderate levels of burnout.

#### Differential analysis of factors influencing burnout among prison police

The differences between male and female officers in the self-efficacy dimension were not statistically significant; the differences in work years, interpersonal relationships, workload, work environment, whether direct contact the of supervision during supervision, sense of organizational fairness, sense of organizational support, and promotion were statistically significant in each dimension. Very bad interpersonal relationships were more likely to produce burnout; workload of ‘very tired and bored' were more likely to result in all dimensions of burnout; work environment of ‘very bad' was more likely to result in emotional exhaustion; and whether direct contact the of supervision during supervision was more likely to lead to emotional exhaustion and negative detachment (Table 5).

**Table 5:** Single-factor analysis of burnout among prison police.

**Factors**	**Group**	**Emotional exhaustion**	**Negative detachment**	**Self-efficacy**
Gender	Male	7.95 ± 3.85	6.61 ± 3.29	26.17 ± 4.18
	Female	6.45 ± 4.57	5.30 ± 3.69	26.13 ± 5.50
	*T*-value	5.479	5.729	0.121
	*P*–value	0.000	0.000	0.904
Working years	<1year	4.83 ± 4.96	3.30 ± 3.83	28.19 ± 3.71
	1–10 years	8.16 ± 4.67	6.08 ± 3.57	25.64 ± 4.66
	10–20 years	7.04 ± 3.67	5.77 ± 3.02	25.09 ± 5.52
	20–30 years	7.39 ± 3.62	6.94 ± 3.26	26.56 ± 4.01
	≥30 years	6.85 ± 3.99	6.66 ± 3.52	28.33 ± 3.68
	*F*–value	8.942	13.247	12.356
	*P*–value	0.000	0.000	0.000
Interpersonal relationships	Very bad	15.00 ± 0.00	11.50 ± 0.71	25.50 ± 10.61
	Comparatively bad	11.25 ± 4.27	8.00 ± 3.37	20.00 ± 6.92
	General	9.71 ± 3.53	8.03 ± 2.92	23.05 ± 5.19
	Comparatively good	7.66 ± 3.80	6.53 ± 3.35	26.31 ± 4.24
	Very good	5.91 ± 4.25	4.67 ± 3.30	27.71 ± 3.90
	*F*–value	32.446	37.089	37.711
	*P*–value	0.000	0.000	0.000
Workload	Very tired and bored	14.17 ± 4.06	11.03 ± 3.14	23.00 ± 6.08
	More tired and bored	9.33 ± 2.26	7.77 ± 2.362	25.21 ± 3.02
	General	8.25 ± 2.88	6.56 ± 2.61	24.50 ± 5.26
	More relaxed	3.73 ± 2.38	3.30 ± 2.14	29.36 ± 4.14
	Very easy	0.77 ± 2.06	1.08 ± 1.85	31.12 ± 2.60
	*F*–value	442.315	290.761	96.745
	*P*–value	0.000	0.000	0.000
Working environment	Very poor	17.07 ± 4.01	13.36 ± 3.13	24.29 ± 8.78
	Comparison poor	9.15 ± 2.95	7.46 ± 2.89	24.87 ± 3.78
	General	8.32 ± 3.17	6.87 ± 2.78	25.24 ± 4.33
	Comparatively good	3.91 ± 4.10	3.42 ± 3.28	29.09 ± 4.25
	Very good	2.13 ± 4.57	1.87 ± 4.22	31.60 ± 3.16
	*F*–value	124.566	98.95	46.019
	*P*–value	0.000	0.000	0.000
Whether direct contact with the object of supervision	Direct contact	8.77 ± 3.04	7.27 ± 2.78	25.50 ± 3.80
	Indirect contact	4.48 ± 4.77	3.68 ± 3.62	27.68 ± 5.90
	*T*-value	17.264	17.235	−7.079
	*P*-value	0.000	0.000	0.000
Sense of organizational fairness	Very unfair	13.14 ± 6.45	9.71 ± 6.15	22.10 ± 10.36
	Comparison unfair	9.01 ± 3.18	8.49 ± 2.73	25.22 ± 3.52
	General	8.36 ± 3.58	6.91 ± 2.89	25.29 ± 4.71
	Comparative fairness	6.04 ± 4.09	4.77 ± 3.24	27.28 ± 4.11
	Very fair	3.25 ± 5.14	2.42 ± 4.70	31.17 ± 2.69
	*F*-value	41.263	57.625	20.499
	*P*-value	0.000	0.000	0.000
Sense of organizational support (group building)	Very little	10.26 ± 5.67	7.71 ± 4.58	26.46 ± 6.58
	Less	7.11 ± 3.08	6.47 ± 3.17	26.16 ± 4.19
	General	7.84 ± 4.16	6.36 ± 3.37	25.31 ± 4.86
	Compare more	6.68 ± 4.17	5.45 ± 3.40	27.21 ± 3.87
	Very much	2.67 ± 3.786	1.00 ± 1.00	33.00 ± 2.65
	*F*-value	12.942	9.134	9.310
	*P*-value	0.000	0.000	0.000
Promotion	Very dissatisfied	11.45 ± 6.85	8.05 ± 6.07	25.55 ± 9.02
	More dissatisfied	9.22 ± 3.57	8.78 ± 2.99	25.61 ± 3.87
	General	9.40 ± 3.09	7.62 ± 2.75	24.25 ± 5.32
	More satisfied	7.41 ± 3.44	5.99 ± 2.81	26.10 ± 3.67
	Very satisfied	3.43 ± 3.81	2.96 ± 3.12	29.39 ± 3.38
	*F*-value	96.006	94.663	40.720
	*P*-value	0.000	0.000	0.000

### Analysis of influencing factors

#### Correlation analysis

This study used a single composite measure of burnout in the correlation analysis. Correlation analysis was conducted using eight influencing factors at the work, organizational, and individual levels, to examine the correlation between the three levels and the overall burnout status. Therefore, the total burnout score was used as a single composite measure of burnout. We used bivariate analysis to evaluate the correlation between eight questions in the three influencing factors (Table 6). Correlation coefficients of the eight questions were workload: −0.539 (*p* = 3.495E-78), work environment: 0.341 (*p* = 3.1572E-29), sense of rejection: −0.489 (*p* = 1.4965E-62), sense of fairness: 0.273 (*p* = 5.6024E-19), number of reunions: 0.124 (*p* = 0.000069), treatment/promotion: 0.350 (*p* = 6.6688E-31), interpersonal relationship: 0.157 (*p* = 4.3863E-7), and physical condition: 0.460 (*p* = 1.0084E-54), which had upper significant correlations at the 0.01 (two-sided) level. Table 7 reveals that the absolute value of Pearson's coefficient of correlation for workload was the largest, which indicated that workload had the strongest correlation with burnout, followed by the physical condition and sense of rejection.

**Table 6:** Correlation analysis of influencing factors and burnout.

**Burnout**	**Sig. (bilaterally)**		
@1 Workload	Pearson correlation	−0.539^**^	0.000
@2 Working environment	Pearson correlation	0.341^**^	0.000
@3 Sense of rejection	Pearson correlation	−0.489^**^	0.000
@4 Sense of fairness	Pearson correlation	0.273^**^	0.000
@5 Number of group building	Pearson correlation	0.124^**^	0.000
@6 Promotion	Pearson correlation	0.350^**^	0.000
@7 Interpersonal relationships	Pearson correlation	0.157^**^	0.000
@8 Physical condition	Pearson correlation	0.460^**^	0.000

a.

** Significantly correlated at the 0.01 level (bilaterally). b. List *N* = 1,024.

**Table 7:** Multiple linear regression analysis of three dimensions of burnout among prison officers.

**Model**	**Non-standardized coefficient**		**Standard coefficient**	***t*-value**	**Sig**.	**R-side (adjusted R-side)**	***F***	
	**B**	**Standard error**						
1	(Constant)	3.994	0.960		4.162	0.000	0.729 (0.728) Durbin-Watson = 1.678	913.173 (*P* = 0.000^a^)
	Work level	0.464	0.119	0.076	3.887	0.000		
	Organization level	2.555	0.097	0.507	26.240	0.000		
	Personal level	3.956	0.198	0.422	19.982	0.000		

a Dependent variable: total score of burnout.

#### Multiple linear regression analysis

Table 7 shows that the adjusted R-square reached 72.8%, thus indicating that 72.8% of the change in the burnout level could be explained by the independent variables. The adjusted R-square value >50% indicated that this data had research significance. Furthermore, a Durbin-Watson value of 1.678, which was closer to 2, revealed that this model had better independence in the sample data. *P* = 0.000 denotes that at least one of the independent variables in this study can significantly affect the dependent variable, burnout. To further determine the independent variables, it is necessary to analyze the coefficient table. As can be seen, *P* = 0.000 for the work, organizational, and personal levels suggests that these factors can significantly affect the degree of burnout. All of the control variables can significantly affect the dependent variable, burnout, thereby further illustrating the necessity of including control variables in this model. The coefficients of influence of work level, organizational level, and personal level on burnout are 0.076, 0.507, and 0.422, respectively; all of which are >0 indicating that organizational, job, and personal factors have a significant positive influence on burnout.

The total score of the three dimensions of burnout was used as the dependent variable, each factor was selected as an independent variable, and dummy variables were set for the independent variables with two or more groups. Each reference group was selected for multiple linear pre-regression, and variables with multiple covariances were excluded (Table 8). The results showed that gender, years of work experience, workload, direct contact with the criminals during supervision, and sense of organizational support did not have multiple covariances and could be included in the final multiple linear analysis models. The regression results showed that: there was a significant correlation between gender and burnout and the burnout of women was less than that of men; there was no significant correlation between years of work and burnout (*P* = 0.136); there was a significant correlation between workload and burnout and the burnout of those who were “very tired” was significantly higher than that of those who were “more tired and relaxed” at work; there was a significant correlation between having whether direct contact the of supervision during supervision and burnout and burnout was higher among prison officers with whether direct contact the of supervision during supervision; there was a significant correlation between organizational support and burnout was higher among those with “very little” group building activities than those with “very much and more” group building activities.

**Table 8:** Multiple linear regression analysis of specific factors influencing burnout among prison officers.

**Factors**	**Reference group**	**Non-standardized coefficient (b)**	**Standard deviation of the sample mean ($S\bar{\text{x}}$)**	***t*-value**	***P*-value**	**Standardized coefficient (*b^′^*)**	***VIF***
Gender	Male	−2.853	0.435	−6.554	0.000	−0.201	1.000
Years of work	<1 year	1.580	1.060	1.491	0.136	0.092	4.094
Interpersonal Relationships	Very bad	−11.216	4.650	−2.412	0.016	−0.657	78.170
Workload	Very tired	−5.881	0.966	−6.087	0.000	−0.441	7.586
Working Environment	Very bad	−13.226	1.689	−7.831	0.000	−0.801	12.592
Whether direct contact with the object of supervision	Direct contact	−5.695	0.415	−13.711	0.000	−0.394	1.000
Sense of organizational support	Very little	−4.683	0.915	−5.117	0.000	−0.298	3.577
Sense of organizational fairness	Very unfair	−4.390	1.432	−3.065	0.002	−0.323	12.227
Promotion	Very dissatisfied	−5.539	1.357	−4.082	0.000	−0.412	11.922

## Discussion

Burnout among police has been increasingly receiving academic attention. Existing studies have explored ways to alleviate the burnout from the perspective of work-family conflict and de-escalation (41). Studies have pointed out that emotional labor and value dissonance increase the level of burnout (42). In addition to the Maslach Burnout Questionnaire used in this study, the Granada Burnout Questionnaire, pretested self-administered questionnaire (SAQ), and the SBI (Spanish Burnout Inventory) questionnaire have been utilized in different studies to evaluate burnout among police (43–45). Prison police officers are engaged in the long-term monotonous and boring work of reforming criminals where it is difficult to receive immediate results, which inevitably leads to a high level of burnout among the officers (46). This study shows that the burnout of prison police is high and there are different levels of burnout in three dimensions: emotional exhaustion, negative detachment, and self-efficacy. Moreover, the results of multiple linear regression analysis show that gender, workload, whether direct contact with the object of supervision, and sense of organizational support significantly affect the degree of burnout and the influencing factors can be categorized into three levels: work, organizational and personal.

Analysis of the influencing factors of burnout shows that workload, work environment, and direct contact with criminals have a great influence on the level of burnout. Possible reasons for this are that prison police generally have long working hours and a heavy workload, and those who have direct contact with the object of supervision have a heavier workload. Prison police work in an environment which is isolated from the world by high walls and power grids. Many prisons are engaged in garment production and processing work, which results in a lot of dust and particles in the air, which can easily have an impact on the health of prison police. Prison officers also face risks in law enforcement, such as escapes and physical assaults. Frequent contact with criminals also makes the officers susceptible to the influence of negative thinking about criminals, which makes them bear a huge psychological pressure easily leading to burnout. Wickramasinghe pointed out that the greater the workload, the higher the degree of burnout of prison officers (47). Excessive work pressure on prison police officers leads to longer working hours, higher workload, and greater work intensity. They face a more closed work environment, the work content is boring, the atmosphere is dull and monotonous, and the number of psychological and behavioral abnormalities in criminals is high. The greater the workload, the higher the burnout of prison police who feel very tired and bored (48). The study shows that the level of burnout is higher for prison officers who have direct contact with the object of supervision. The reason for this is that prison officers who have direct contact with the object of supervision have long working hours, high pressure, often work overtime, and cannot take care of their families, which makes them seriously question the value and meaning of their work. Whereas, prison officers who have indirect contact with the object of supervision have relatively short working hours and their work is relatively easy (3).

Analysis of the influencing factors at the organizational level shows that many prison police officers believe that there is inequity in treatment and job promotion at their workplace. The possible reason is that some prison police officers believe that there is not much correlation between workload and pay and that their salary is unaffected by the amount of work they do (33). The workload is heavy, but there is little time for rest. There are also many prison police officers who are not satisfied with the promotion. They are not satisfied with the promotions they receive at work, there are no opportunities for promotion or they do not receive enough extra compensation after promotion, etc. All these impact on their motivation at work by varying degrees and this negative work attitude can easily lead to burnout (49). The sense of support at the organizational level, i.e., whether the unit often organizes group building activities, etc., also affects the degree of burnout. The first dimension of burnout of prison officers is emotional exhaustion, i.e., they are not energetic and enthusiastic at work and often feel that they are in a tired state. If the prison regularly organizes group building activities to make everyone happy physically and mentally, work efficiency will improve (50). The second dimension is negative detachment, which results in individuals distancing themselves from work and adopting a negative and indifferent attitude toward it. If prisons improve their incentive structures and assessment systems for the officers, their attitude toward work will be more positive, they will also has something to do with recognizing their abilities and using them in the workplace and improve their sense of self-achievement. This idea is similar to previous studies that indicate that police burnout is associated with negative emotions and also indicate post-traumatic stress disorder as a factor (3).

From the analysis of influencing factors at the personal level, we can see that due to a heavy workload, frequent overtime, and night duty, prison police do not have days off and holidays. When their friends, engaged in other jobs, use weekends to gather and go on trips, prison police may be busy at their work and do not have much time to communicate with friends and family. The only people they come in contact with at work are colleagues and criminals, making their social circle narrow. Heavy workload and psychological stress can easily affect the physical and mental health of prison officers which in turn affects their burnout level. At the same time, there is more burnout among men than among women prison police, probably because only two women's prisons were investigated and female officers in men's prisons are not allowed to come in contact with the object of supervision. Therefore, there are far fewer female prison police than their male counterparts, and most female officers are engaged in work that does not involve direct contact with the object of supervision. So, their work pressure is low, and they work less overtime and fewer night shifts, which greatly reduces burnout. Li and Liu pointed out that burnout among prison officers was influenced by gender and was significantly higher among male officers (51). Veljković also pointed out that burnout of prison police was affected by gender, age, etc. (6). All of these are consistent with the findings of this investigation.

The Pearson correlation coefficient of the number of reunions had the smallest absolute value, thus indicating that the number of reunions had the weakest correlation with burnout. Therefore, in the future improvement of burnout among prison police officers, priority should be given to factors such as workload, physical condition, sense of rejection, treatment promotion, and work environment. Furthermore, secondary consideration should be given to factors such as sense of fairness, interpersonal relationships, and the number of reunion building. The significance (two-sided) for each factor was correlated at 0.01 level. It indicates that eight aspects, including work level, organization level and personal level, are significantly correlated with burnout, while workload and exclusion are significantly negatively correlated with burnout (The reason for the negative correlation is related to the presence of reverse scoring in the Likert scale: in workload, a score of 1 was assigned for very tired and bored and a score of 5 for very relaxed; in sense of rejection, a score of 1 was assigned for very strong repulsion and a score of 5 for no repulsion). Previous research has shown that personal factors such as experiencing victimization and greater job demands were associated with more stress for prison officers (7).Task-oriented coping was positively associated with personal achievement. Social support at work is negatively associated with emotional exhaustion (52). The difference between our study and previous research correlations is due to the assigned scores, but the findings are the same: the more fatigued the work (Lower the score), the stronger the burnout; the stronger the exclusion (Lower the score), the more pronounced the burnout.

## Conclusions

Prison police are an important part of society, especially as they are responsible for maintaining the law and order and social stability. Because of the unique nature of their job, prison police are prone to high levels of burnout which affects their physical and mental health, and makes them less motivated and efficient at work. This study explores the current state of burnout among prison police through a survey using a well-established questionnaire. The findings point out the dimensions and influencing factors of burnout in prison police. The study also provides recommendations based on the findings to improve this. Hence, the study is relevant and its contributions are significant. Through the above analysis at the personal, work, and organizational levels, it is found that the burnout of prison officers should be reduced through preferential treatment of prison officers, sound organizational mechanisms, and self-improvement of prison officers.

### Preferential treatment

At the work level, prisons should, according to the actual situation, establish a work and rest system suitable for front-line prison officers and an economic compensation system for their overtime, pay real attention to their health and work situation, solve their difficulties in time, and reduce the negative impact resulting from prison work, so that the officers feel that their organization cares for them and will work with enthusiasm, thus improving work efficiency. It has been found that the difference between contributions and rewards observed in the workplace had a significant impact on burnout among prison officers (53).

### Sound organizational mechanism

At the organizational level, first, a fair and reasonable selection and promotion system should be established. This can not only effectively motivate all types of personnel to make best use of their talents, but also promote better development of the prison. Second, the performance evaluation mechanism should be fair and effective. The different positions and duties of prison police should be analyzed, a scientific and reasonable performance assessment mechanism should be formulated, regular assessments of the performance should be conducted, and the scope of responsibility and the amount of duties of each prison police official should be clarified. At the same time, a performance assessment supervision mechanism should be established to ensure that the performance assessment is fair, just, and open. Conducting performance appraisals for different positions and posts can be an important basis for the promotion and adjustment of prison police positions, and can be useful in designing policies and programs in support of recruitment and management training, and police counseling services (54).

### Self-improvement

At the personal level, long-term accumulation of bad emotions has a negative impact on people's physical and mental health so that people's physical immunity decreases, work motivation reduces and, in serious cases, physical or psychological problems may occur. When a person is in a bad mood, finding someone to talk to can help to reduce the bad mood and relieve psychological pressure. Prison police should participate in more physical health activities after work (55), cultivate the interest of life while improving their attitude toward life, maintain work-life balance, and strive to create a harmonious family atmosphere, practice introspection (where you work to understand yourself), secure and perform work they enjoy, learn to find pleasure in ordinary work, and maintain a positive attitude to work. This will help address the contemporary challenges of police work and develop psychological skills (56).

### Limitations of the study

This study has some limitations. First, it only demonstrates the burnout status and its influencing factors among prison police at present, and it is difficult to monitor past and future dynamic changes. In terms of sample selection, this study only investigated prison police in Liaoning Province, China, and their burnout status may not be the same in other regions of China, so the conclusions may not be suitable for generalization. Second, the questionnaire used in the study is, to some extent, influenced by individual perceptions and the local social environment, and inferences about the causal relationships between variables need to be strengthened in the future. It is expected that a combination of quasi-experimental and value-based research will be used to track the long-term process of dynamic changes in burnout among prison police officers, to more clearly show the developmental process and causal mechanisms of burnout.

## Data availability statement

The original contributions presented in the study are included in the article/Supplementary material, further inquiries can be directed to the corresponding author/s.

## Author contributions

JG: conceptualization, methodology, software, investigation, formal analysis, and writing-original draft. XD: data curation and writing-original draft. QG: conceptualization, resources, supervision, and writing-review and editing. All authors contributed to the article and approved the submitted version.

## Conflict of interest

The authors declare that the research was conducted in the absence of any commercial or financial relationships that could be construed as a potential conflict of interest.

## Publisher's note

All claims expressed in this article are solely those of the authors and do not necessarily represent those of their affiliated organizations, or those of the publisher, the editors and the reviewers. Any product that may be evaluated in this article, or claim that may be made by its manufacturer, is not guaranteed or endorsed by the publisher.

## References

[1] Queirós C Passos F Bártolo A Faria S Fonseca SM Marques AJ. Job stress, burnout and coping in police officers: relationships and psychometric properties of the organizational police stress questionnaire. Int J Environ Res Public Health. (2020) 17:6718. 10.3390/ijerph17186718 32942672 PMC7557776

[2] Patel R Huggard P Toledo A. Occupational stress and burnout among surgeons in Fiji. Front Public Health. (2017) 5:41. 10.3389/fpubh.2017.00041 28337433 PMC5343003

[3] Juczyński Z Ogińska-Bulik N. Ruminations and occupational stress as predictors of post-traumatic stress disorder and burnout among police officers. Int J Occup Safety Ergon. (2022) 28:743–50. 10.1080/10803548.2021.1907986 33754945

[4] Henry VE. Death Work: Police, Trauma, and the Psychology of Survival. New York, NY: Oxford University Press (2004). p. 158–159.

[5] Akl A Mohiyaldeen I Alshatti R Alenezi O Dougherty R Al-Raihan A. The prevalence of burnout and its associated factors among surgical specialists in kuwait ministry of health hospitals. Front Public Health. (2022) 10:679834. 10.3389/fpubh.2022.679834 35174119 PMC8841660

[6] Rancic DR Mirkovi MR Kuli LM Stankovi VV Stefanovi LS. Burnout among private security staff in serbia: a multicentic cross-sectional study. Front Public Health. (2021) 9:622163. 10.3389/fpubh.2021.622163 34079781 PMC8166244

[7] Steiner B Wooldredge J. Individual and environmental sources of work stress among prison officers. Crim Justice Behav. (2015) 42:800–18. 10.1177/009385481456446316012372

[8] Burke RJ Mikkelsen A. Burnout, job stress and attitudes towards the use of force by Norwegian police officers. Polic Internat J Polic Strat Manag. (2005) 28:269–78. 10.1108/13639510510597906

[9] Freudenberger HJ. Staff Burn-out. Journal of Social Issues. (1974) 30:159–65. 10.1111/j.1540-4560.1974.tb00706.x

[10] Maslach C. Job burnout: new directions in research and intervention. Curr Dir Psychol Sci. (2003) 12:189–92. 10.1111/1467-8721.01258

[11] Pines A Aronson E. Career Burnout: Causes and Cures. Neuva york, Free Press (1988). p. 243–246.

[12] Leiter MP. Coping patterns are predictors of burnout: the function of control and escapist coping patterns. J Organiz Behav. (1991) 12:123–44. 10.1002/job.4030120205

[13] Hobfoll SE. Conservation of resources: a new attempt at conceptualizing stress. Am Psychol. (1989) 44:513–24. 10.1037/0003-066X.44.3.513 2648906

[14] Baldwin S Bennell C Andersen JP Semple T Jenkins B. Stress-activity mapping: physiological responses during general duty police encounters. Front Psychol. (2019) 10:2216. 10.3389/fpsyg.2019.02216 31636582 PMC6788355

[15] Maslach C Goldberg J. Prevention of burnout: New perspectives. Appl Prev Psychol. (1998) 7:63–74. 10.1016/S0962-1849(98)80022-X

[16] Aljabri D Alshatti F Alumran A Al-Rayes S Alsalman D Althumairi A. Sociodemographic and occupational factors associated with burnout: a study among frontline healthcare workers during the COVID-19 pandemic. Front Public Health. (2022) 10:854687. 10.3389/fpubh.2022.854687 35356019 PMC8959574

[17] Carbonneau N Vallerand RJ Fernet C Guay F. The role of passion for teaching in intrapersonal and interpersonal outcomes. J Educ Psychol. (2008) 100:977–87. 10.1037/a0012545

[18] Karasek R. Job demands, job decision latitude, and mental strain: Implications for job redesign. Administ Sci Quart. (1979) 24:285–308. 10.2307/2392498

[19] Johnson JV Hall EM. Job strain, work place social support and cardiovascular disease: A cross sectional study of a random sample of the Swedish working population. Am J Public Health. (1988) 78:1336–42. 10.2105/AJPH.78.10.1336 3421392 PMC1349434

[20] Liu Y Li FF. Research progress of burnout among foreign special education teachers. Spec Educ China. (2016) 9:65–71. 10.3969/j.issn.1007-3728.2016.09.011

[21] Morgan RD Van Haveren RA Pearson C. Correctional officer burnout. Further Anal Crim Just Behav. (2002) 29:144–60. 10.1177/0093854802029002002

[22] Kop N Euwema M Schaufeli W. Burnout, job stress and violent behaviour among dutch police officers. Work Stress. (1999) 13:326–40. 10.1080/02678379950019789

[23] Euwema MC Kop N Bakker AB. The behavjour of police officers in conflict situations: how burnout an reduced dominance contribute to better outcomes. Work Stress. (2014) 18:23–38. 10.1080/0267837042000209767

[24] Yun I Hwang E Lynch J. Police stressors, job satisfaction, burnout, and turnover intention among south korean police officers. Asian Criminol. (2015) 10:23–41. 10.1007/s11417-015-9203-4

[25] Smoktunowicz E Baka L Cieslak R Nichols CF Benight CC Luszczynska A. Explaining counterproductive work behaviors among police officers: the indirect effects of job demands are mediated by job burnout and moderated by job control and social support. Human Performance. (2015) 28:332–50. 10.1080/08959285.2015.1021045

[26] Basinska BA Wiciak I Daderman AM. Fatigue and burnout in police officers: the mediating role of emotions. Polic Int J Polic Strat Manag. (2014) 37:665–80. 10.1108/PIJPSM-10-2013-0105

[27] Queirós C Passos F Bártolo A Marques AJ da Silva CF Pereira A. Burnout and stress measurement in police officers: literature review and a study with the operational police stress questionnaire. Front Psychol. (2020) 11:587. 10.3389/fpsyg.2020.00587 32457673 PMC7221164

[28] Carlson J Thomas G. Burnout among prison caseworkers and corrections officers. J Offender Rehabil. (2006) 43:19–34. 10.1300/J076v43n03_02

[29] Tyagi A Dhar RL. Factors affecting health of the police officials: mediating role of job stress. Polic Int J Polic Strat Manag. (2014) 37:649–64. 10.1108/PIJPSM-12-2013-0128

[30] Tewksbury R Higgins GE. Prison staff and work stress: the role of organizational and emotional influences. Am J Crim Just. (2006) 30:247–66. 10.1007/BF02885894

[31] Lambert EG Hogan NL Griffin ML. The impact of distributive and procedural justice on correctional staff job stress, job satisfaction, and organizational commitment. J Crim Justice. (2007) 35:644–56. 10.1016/j.jcrimjus.2007.09.001

[32] Armstrong G Griffin M. Does the job matter? Comparing correlates of stress among treatment and correctional staff in prisons. J Crim Just. (2004) 32:577–92. 10.1016/j.jcrimjus.2004.08.007

[33] Garland B. The impact of administrative support on prison treatment staff burnout: an exploratory study. Prison J. (2004) 84:452–71. 10.1177/0032885504269343

[34] Backteman-Erlanson S Padyab M Brulin C. Prevalence of burnout and associations with psychosocial work environment, physical strain, and stress of conscience among Swedish female and male police personne. Polic Pract Res. (2012) 14:491–505. 10.1080/15614263.2012.736719

[35] Portoghese I Leiter MP Maslach C Galletta M Porru F D'Aloja E. Measuring burnout among university students: factorial validity, invariance, and latent profiles of the italian version of the maslach burnout inventory student survey (MBI-SS). Front Psychol. (2018) 9:2105. 10.3389/fpsyg.2018.02105 30483171 PMC6240654

[36] Maslach C Wilmar B Schaufeli, Michael P. Job Burnout Ann Rev Psychol. (2001) 52:397–422. 10.1146/annurev.psych.52.1.397 11148311

[37] Schutte N Toppinen S Kalimo R Schaufeli W. The factorial validity of the Maslach Burnout Inventory-General Survey (MBI-GS) across occupational groups and nations. J Occup Organ Psychol. (2000) 73:53–66. 10.1348/096317900166877

[38] Cortez AR Winer LK Kassam AF Hanseman DJ Kuethe JW Sussman JJ. Exploring the relationship between burnout and grit during general surgery residency: a longitudinal, single-institution analysis. Am J Surgery. (2020) 219:322–7. 10.1016/j.amjsurg.2019.09.041 31623881

[39] Joaquim A Custódio S Savva-Bordalo J Chacim S Carvalhais I Lombo L. Burnout and occupational stress in the medical residents of oncology, haematology and radiotherapy: a prevalence and predictors study in portugal, psychology. Health Med. (2017) 23:317–24. 10.1080/13548506.2017.1344256 28661187

[40] Shrestha N. Factor analysis as a tool for survey analysis. Am J Appl Mathem Stat. (2021) 9:4–11. 10.12691/ajams-9-1-215330692

[41] Griffin JD Sun IY. Do work-family conflict and resiliency mediate police stress and burnout: a study of state police officers. Am J Crim Just. (2018) 43:354–70. 10.1007/s12103-017-9401-y

[42] Schaible LM Gecas V. The impact of emotional labor and value dissonance on burnout among police officers. Polic Quart. (2010) 3:316–41. 10.1177/1098611110373997

[43] Emilia I Ortega-Campos E Vargas-Roman K Gustavo R Cañadas F Tania Ariza C. Study of the predictive validity of the burnout Granada questionnaire in police officers international. J Environ Res Public Health. (2020) 17:6112. 10.3390/ijerph17176112 32842582 PMC7504042

[44] Wickramasinghe ND Wijesinghe PR. Burnout subtypes and associated factors among police officers in Sri Lanka: a cross-sectional study. J Forensic Leg Med. (2018) 58:192–8. 10.1016/j.jflm.2018.07.006 30015221

[45] Gil-Monte PR Carretero N Rold MD NÚÑEz-RomEM. Burnout prevalence amongst instructors of disabled people. J Work Organiz Psychol. (2005) 21:107–23. Available online at: https://journals.copmadrid.org/jwop/files/96810.pdf (accessed March 15, 2022).

[46] Zou CH Liu XL. Revision of the genus phyllostachys spp. A study on the relationship between burnout and social support personality characteristics of prison police officers. J Chaohu Coll. (2010) 1:30–4. 10.3969/j.issn.1672-2868.2010.01.007

[47] Gil-Monte PR Figueiredo-Ferraz H Valdez-Bonilla H. Factor analysis of the Spanish Burnout Inventory among Mexican prison employees. Can J Behav Sci. (2013) 2013:417-24. 10.1037/a0027883

[48] Montero-Marín J García-Campayo J Fajó-Pascual M Carrasco JM Gascón S Gili M. Sociodemographic and occupational risk factors associated with the development of different burnout types: the cross-sectional University of Zaragoza study. BMC Psychiatry. (2011) 11:49. 10.1186/1471-244X-11-49 21447169 PMC3074532

[49] Schiff M Leip L. The impact of job expectations, workload, and autonomy on work-related stress among prison wardens in the United States. Crim Justice Behav. (2018) 46:136–53. 10.1177/0093854818802876

[50] Lambert EG Hogan NL Dial KC Jiang S Khondaker MI. Is the job burning me out? An exploratory test of the job characteristics model on the emotional burnout of prison. Staff Prison J. (2011) 92:3–23. 10.1177/0032885511428794

[51] Li HM Liu LQ. An analysis of the current situation of burnout in prison police and its influencing factors. J Guangxi Polic Acad. (2018) 1:36–9. 10.19736/j.cnki.gxjcxyxb.2018.01.007

[52] Cieslak R Korczynska J Strelau J Kaczmarek M. Burnout predictors among prison officers: The moderating effect of temperamental endurance. Pers Individ Dif. (2008) 45:666–72. 10.1016/j.paid.2008.07.012

[53] Isenhardt A. Hostettler U. Inmate violence and correctional staff burnout. J Int Viol. (2016) 35:173–207. 10.1177/0886260516681156 27923874

[54] Loo R. A typology of burnout types among police managers. Polic Int J Polic Strat Manag. (2004) 27:156–65. 10.1108/13639510410536797

[55] García-Rivera BR Olguín-Tiznado JE Aranibar MF Ramírez-Barón MC Camargo-Wilson C López-Barreras JA. Burnout syndrome in police officers and its relationship with physical and leisure activities. Int J Environ Res Public Health. (2020) 15:5586. 10.3390/ijerph17155586 32756344 PMC7432764

[56] Ellrich K. Burnout and violent victimization in police officers: a dual process model. Polic Int J. (2016) 39:652–66. 10.1108/PIJPSM-10-2015-0125

