# Supplementary material for: Analysis of burnout and its influencing factors among prison police
Source: Front Public Health. 2022 Sep 13;10:891745. doi: 10.3389/fpubh.2022.891745 (PMC9513513; doi:10.3389/fpubh.2022.891745)
Supplement: Supplementary file 1 [file Data_Sheet_1.PDF]

Appendix 1: Questionnaire and Sample Information

1 Selection and Characteristics of the Sample

This paper takes the prison police in China as the research object and focuses on the current situation of burnout and the influencing factors of prison police in China. The questionnaire was randomly distributed throughout the prison system in China to ensure the authenticity and reliability of the sample data to the greatest extent. The relationship between demographic variables such as gender, marital status, age, years of work, Police entry method, Positions, education level, Personal monthly income, Whether direct contact the of supervision and Current overtime or night shifts at work, and burnout was studied. See TableA.

Table A. Sample Demographics

Serial number	Variables	Category	Number of people	Percentage %
1	Gender	Male	694	67.77
		Female	330	32.23
2	Marital Status	Unmarried	198	19.34
		Married	797	77.83
		Divorce	29	2.83
3	Age	20-30	330	32.23
		30-40	281	27.44
		40-50	202	29.49
		50-60	122	10.84
4	Years of work	Less than 1 year	47	4.59
		1 year - 10 years	409	39.94
		10 years - 20 years	190	18.55
		20 - 30 years	282	27.54
		Over 30 years	96	9.38
5	Police entry method	Police Academy Graduates	394	38.48
		Social recruitment of non- police academy	340	33.2
		Military transfer cadres	57	5.57
		Others (reassignment to full-time positions, etc.)	233	22.75

6	Positions	Section Member	518	50.59
		Deputy Section Level	331	32.32
		Full Section Level	146	14.26
		Deputy Division	18	1.76
		Positive Division	11	1.07
7	Education Level	Junior high school and below	1	0.1
		High school (including vocational high school)	22	2. 15
		College	283	27.64
		Undergraduate	696	67.97
		Graduate student and above	22	2. 15
8	Personal monthly income	4000RMB-6000RMB	410	40.04
		6000RMB-8000RMB	390	38.09
		8000RMB - 10000RMB	213	20.8
		10000RMB or more	11	1.07
9	Whether direct contact the of supervision	Direct contact	713	69.63
		Indirect Contact	311	30.37
10	Current overtime or night shifts at work	Never	38	3.71
		Occasionally	400	39.06
		Frequently	586	57.23

Data source: information from the questionnaire of 1024 prison police officers in Liaoning prison.

2 Questionnaire design

The questionnaire was divided into three parts, the first part was the basic information on demographic variables, including 10 questions, the second part was the burnout survey of prison police officers including 20 questions, of which 1 to 5 was the emotional exhaustion dimension, 6 to 12 was the negative detachment dimension, and 13 to 20 was the self-efficacy dimension. The third part is the factors influencing burnout of prison police officers divided into 8 questions, among which 1 to 3 are work level, 4 to 6 are organizational level, and 7 and 8 are personal level. See Table B.

Table B. Questionnaire of China Prison Police Occupational Burnout and Influencing Factors

Current status of burnout	Emotional exhaustion	1. Work makes me feel physically and mentally exhausted<br>2. I feel exhausted at the end of the day<br>3. Wake up in the morning and having to face the day's work makes me feel very tired<br>4. The whole day's work makes me feel very stressful<br>5. work makes me feel like I'm going to collapse
	Negative detachment	6. I'm not as interested in my work as I used to be<br>7. I am not as enthusiastic as before in my work<br>8. I tend to become irritable at work<br>9. I care less and less whether my work is meaningful or not<br>10. I feel doubtful about the work I am doing<br>11. I feel that I can't make any progress if I work harder
	Self-efficacy	12.I like what I do<br>13.I feel that my work is valuable<br>14. I am good at my job<br>15. I can do all the work effectively<br>16. I feel happy when I finish some work<br>17. I have made a positive impact on my colleagues through my work<br>18. With colleagues, I can liven up the atmosphere<br>19. I can control my emotions well at work<br>20. I feel that I am making a valuable contribution to the unit
Burnout influencing factors	Work level	1. Do you feel tired and bored with your work?<br>2. Do you think you have a good working environment?<br>3. Do you have a strong sense of rejection when dealing with your supervisors?
	Organizational level	4. Do you think the unit's evaluation of merit and advanced is fair?<br>5. How many times do you organize group activities in your unit?<br>6. Are you satisfied with the benefits and job promotion of your unit?
	Personal level	7. Do you think you have good interpersonal relationships?<br>8. Your current physical condition to adapt to regulatory work?

Demographics	1. Gender<br>2. Marital status<br>3. Age<br>4. Years of work<br>5. The way to join the police<br>6. Positions<br>7. Literacy<br>8. Personal monthly income<br>9. Do you have direct contact with the target of supervision<br>10. You are currently working overtime or night shifts at work

Questionnaire on burnout and influencing factors of prison police in Liaoning Province

Appendix 2 Tables in Manuscript

Table 1. Cronbach′s Alpha Coefficients for Subscales and Total Scales

Dimension Name	Cronbach α Confidence Coefficient
Emotional exhaustion	0.906
Negative detachment	0.914
Self-efficacy	0.873
Work level	0.833
Organization level	0.739
Personal level	0.580
The whole questionnaire	0.953

Table 2. KMO and Bartlett′s Test of the Scale

The Kaiser- Meyer- Olkin Metric of Sampling Adequacy		0.952
Bartlett' s sphericity test	Approximate cardinality	22839.776
	df	378
	Sig.	0.000

Table 3. Eigenvalues, Contribution Rates, and Cumulative Contribution Rates of Each Factor

Factors	Initial Eigenvalue			Extraction of Squares and Loading			Rotate Square and Load		
	Total	% of variance	Cumulative %	Total	% of variance	Cumulative %	Total	% of variance	Cumulative %
1Emotional exhaustion	12.705	45.376	45.376	12.705	45.376	45.376	7.085	25.302	25.302
2 Negative detachment	2.602	9.292	54.668	2.602	9.292	54.668	3.586	12.807	38.110
3 Self-efficacy	2.047	7.310	61.977	2.047	7.310	61.977	3.207	11.453	49.563
4 Work dimension	1.560	5.570	67.547	1.560	5.570	67.547	2.793	9.975	59.537
5Organizational dimension	1.181	4.218	71.765	1.181	4.218	71.765	2.361	8.434	67.971
6 Personal dimension	0.815	2.911	74.677	0.815	2.911	74.677	1.878	6.706	74.677

Table 4. Number of Highly Job Burnout in Various Dimensions

Dimensionality	High Burnout		moderate burnout		low burnout	
	Number of people	Percentage	Number of people	Percentage	Number of people	Percentage
Emotional exhaustion	312	30.5%	390	38.1%	321	31.4%
Negative detachment	399	39%	419	41%	307	30%
Self-efficacy	303	29%	460	40.5%	367	30.5%

Table 5. Single-factor Analysis of Burnout Among Prison Police

Factors	Group	Emotional Exhaustion	Negative Detachment	Self-efficacy
Gender	Male	7.95±3.85	6.61±3.29	26.17±4.18
	Female	6.45±4.57	5.30±3.69	26.13±5.50
	t-value	5.479	5.729	0.121
	P-value	0.000	0.000	0.904
Working years	<1year	4.83±4.96	3.30±3.83	28.19±3.71
	1year ~ 10years	8.16±4.67	6.08±3.57	25.64±4.66
	10years ~ 20years	7.04±3.67	5.77±3.02	25.09±5.52
	20years ~ 30years	7.39±3.62	6.94±3.26	26.56±4.01
	⩾30years	6.85±3.99	6.66±3.52	28.33±3.68
	F-value	8.942	13.247	12.356
	p-value	0.000	0.000	0.000
Interpersonal Relationships	Very bad	15.00±0.00	11.50±0.71	25.50±10.61
	Comparatively bad	11.25±4.27	8.00±3.37	20.00±6.92
	General	9.71±3.53	8.03±2.92	23.05±5.19
	Comparatively good	7.66±3.80	6.53±3.35	26.31±4.24
	Very good	5.91±4.25	4.67±3.30	27.71±3.90
	F-value	32.446	37.089	37.711
	p-value	0.000	0.000	0.000
Workload	Very tired and bored	14.17±4.06	11.03±3.14	23.00±6.08
	More tired and bored	9.33±2.26	7.77±2.362	25.21±3.02
	General	8.25±2.88	6.56±2.61	24.50±5.26
	More relaxed	3.73±2.38	3.30±2.14	29.36±4.14
	Very easy	0.77±2.06	1.08±1.85	31.12±2.60
	F-value	442.315	290.761	96.745
	p-value	0.000	0.000	0.000
Working Environment	Very poor	17.07±4.01	13.36±3.13	24.29±8.78
	Comparison poor	9.15±2.95	7.46±2.89	24.87±3.78
	General	8.32±3.17	6.87±2.78	25.24±4.33
	Comparatively good	3.91±4.10	3.42±3.28	29.09±4.25
	Very good	2.13±4.57	1.87±4.22	31.60±3.16
	F-value	124.566	98.95	46.019
	p-value	0.000	0.000	0.000
Whether direct contact with the object of supervision	Direct contact	8.77±3.04	7.27±2.78	25.50±3.80
	Indirect	4.48±4.77	3.68±3.62	27.68±5.90

	Contact			
	t-value	17.264	17.235	-7.079
	P-value	0.000	0.000	0.000
Sense of Organizational fairness	Very Unfair	13.14±6.45	9.71±6.15	22.10±10.36
	Comparison Unfair	9.01±3.18	8.49±2.73	25.22±3.52
	General	8.36±3.58	6.91±2.89	25.29±4.71
	Comparative Fairness	6.04±4.09	4.77±3.24	27.28±4.11
	Very fair	3.25±5.14	2.42±4.70	31.17±2.69
	F-value	41.263	57.625	20.499
	P-value	0.000	0.000	0.000
Sense of Organizational support (group building)	Very little	10.26±5.67	7.71±4.58	26.46±6.58
	Less	7.11±3.08	6.47±3.17	26.16±4.19
	General	7.84±4.16	6.36±3.37	25.31±4.86
	Compare more	6.68±4.17	5.45±3.40	27.21±3.87
	Very much	2.67±3.786	1.00±1.00	33.00±2.65
	F-value	12.942	9.134	9.310
	P-value	0.000	0.000	0.000
Promotion	Very dissatisfied	11.45±6.85	8.05±6.07	25.55±9.02
	More dissatisfied	9.22±3.57	8.78±2.99	25.61±3.87
	General	9.40±3.09	7.62±2.75	24.25±5.32
	More satisfied	7.41±3.44	5.99±2.81	26.10±3.67
	Very satisfied	3.43±3.81	2.96±3.12	29.39±3.38
	F-value	96.006	94.663	40.720
	P-value	0.000	0.000	0.000

Table 6. Correlation Analysis of Influencing Factors and Burnout

Burnout			Sig.(bilaterally)
@1 workload	Pearson correlation	-0.539**	0.000
@2working environment	Pearson correlation	0.341**	0.000
@3sense of rejection	Pearson correlation	-0.489**	0.000
@4sense of fairness	Pearson correlation	0.273**	0.000
@5Number of group building	Pearson correlation	0.124**	0.000
@6 Promotion	Pearson correlation	0.350**	0.000
@7Interpersonal Relationships	Pearson correlation	0.157**	0.000
@8physical condition	Pearson correlation	0.460**	0.000

**. Significantly correlated at the .01 level (bilaterally).

b. List N= 1024

Table 7. Multiple Linear Regression Analysis of Three Dimensions of Burnout Among Prison Officers

Model		Non-standardized Coefficient		Standard Coefficient	t-value	Sig.	R-side (adjusted R-side)	F
		B	Standard Error					
1	(Constant)	3.994	0.960		4.162	0.000	0.729 (0.728) Durbin-Watson=1.678	913.173 (P=0.000b)
	Work level	0.464	0.119	0.076	3.887	0.000		
	Organization level	2.555	0.097	0.507	26.240	0.000		
	Personal level	3.956	0.198	0.422	19.982	0.000		

a. Dependent variable: Total score of burnout

Table 8. Multiple Linear Regression Analysis of Specific Factors Influencing Burnout Among Prison Officers

Factors	Reference Group	non-standardized coefficient (b)	Standard deviation of the sample mean($S_{\bar{x}}$)	t-value	P-value	standardized coefficient (b')	*VIF*
Gender	Male	-2.853	0.435	-6.554	0.000	-0.201	1.000
Years of work	<1 year	1.580	1.060	1.491	0.136	0.092	4.094
Interpersonal Relationships	Very bad	-11.216	4.650	-2.412	0.016	-0.657	78.170
Workload	Very tired	-5.881	0.966	-6.087	0.000	-0.441	7.586
Working Environment	Very bad	-13.226	1.689	-7.831	0.000	-0.801	12.592
Whether direct contact with the object of supervision	Direct contact	-5.695	0.415	-13.711	0.000	-0.394	1.000
Sense of organizational support	Very little	-4.683	0.915	-5.117	0.000	-0.298	3.577
Sense of organizational fairness	Very Unfair	-4.390	1.432	-3.065	0.002	-0.323	12.227
Promotion	Very dissatisfied	-5.539	1.357	-4.082	0.000	-0.412	11.922
